# Dizziness and spatial disorientation in patients with transient global amnesia: clinical and vestibular correlates

**DOI:** 10.1007/s00415-025-13336-1

**Published:** 2025-08-27

**Authors:** Ileok Jung, Jin-Man Jung, Moon-Ho Park, Ji-Soo Kim

**Affiliations:** 1 Department of Neurology, Konkuk University College of Medicine, Konkuk University Medical Center, Seoul, Republic of Korea; 2 Department of Neurology, Korea University College of Medicine, Korea University Ansan Hospital, Ansan, Republic of Korea; 3 Department of Neurology, Seoul National University College of Medicine, 173-82 Gumi-ro, Bundang-gu, Seongnam-si, Gyeonggi-do 13620 South Korea; 4 Dizziness Center, Clinical Neuroscience Center and Department of Neurology, Seoul National University Bundang Hospital, Seongnam, Republic of Korea

**Keywords:** Transient global amnesia, Dizziness, Vertigo, Spatial disorientation, Hippocampus, Vestibular function, Neuropsychological assessment

## Abstract

**Objective:**

To determine the prevalence and clinical significance of dizziness and spatial disorientation in patients with transient global amnesia (TGA), and to examine their relationships with vestibular function and imaging findings.

**Methods:**

We retrospective analyzed 64 TGA episodes from 63 patients [46 (71.9%) women, mean age ± SD = 58.6 ± 9.4]. Vestibular and ocular motor function test [including vestibular-evoked myogenic potentials (VEMPs)], neuropsychological assessments, and brain imaging were evaluated. Logistic regression was used to identify factors associated with dizziness and spatial disorientation.

**Results:**

Dizziness was reported in 30.6% (19/62 episodes), and spatial disorientation in 25.0% (16/64 episodes). In multivariable analysis, dizziness was independently associated with headache [odds ratio (OR) = 6.94; 95% confidence interval (CI) 1.91–25.25; *p* = 0.003] and Valsalva-like provoking events (OR = 4.19; 95% CI 1.14–15.38; *p* = 0.031). Abnormalities in ocular vestibular-evoked myogenic potentials (VEMPs) were significantly association with dizziness in univariate analysis (OR = 4.00; 95% CI 1.13–14.09; *p* = 0.031), but not in the multivariable analysis. In relation to spatial disorientation, abnormal cervical VEMPs showed a significant difference in chi-square test (*p* = 0.043), and a trend toward association in multivariable analysis (OR = 3.28; 95% CI 0.885–12.19; *p* = 0.076), suggesting otolithic vestibular dysfunction. There was a trend-level association between spatial disorientation and lower Korea-Montreal Cognitive Assessment scores (*p* = 0.066). Interestingly, patients in the spatial disorientation group showed better performance in visual memory immediate recall (*p* = 0.061), although this did not reach statistical significance. Hippocampal lesions were identified in 75.0% of TGA episodes, most commonly involving the hippocampal head. However, lesion laterality (left, right, or bilateral) was not associated with dizziness, spatial disorientation, or vestibular abnormalities.

**Conclusion:**

Dizziness and spatial disorientation are common and clinically meaningful symptoms in TGA, likely reflecting transient dysfunction of specific vestibular pathways. These findings suggest broader network involvement in TGA in addition to the hippocampus, particularly implicating otolith-ocular and vestibulo-spinal pathways.

## Introduction

Transient global amnesia (TGA) is characterized by a sudden onset of antero- and retrograde amnesia lasting less than 24 h [1]. During the acute phase, patients may experience mild autonomic or vegetative symptoms such as headache, dizziness, and nausea [2]. Despite extensive clinical and imaging studies, the exact etiology and pathophysiology of TGA remain incompletely understood. Focal lesions involving the hippocampal CA1 region, identifiable on diffusion-weighted MRI (DWI), are the most consistent imaging findings in TGA [3]. Beyond its well-established role in memory, the hippocampus is also involved in processing vestibular and visuospatial information through its integration with multisensory cortical and subcortical circuits [4–7]. These include the pathways associated with spatial navigation, balance, and bodily orientation. Therefore, hippocampal dysfunction during TGA may result not only in memory impairments but also in transient or prolonged deficits in spatial orientation and vestibular processing. In clinical practice, however, atypical presentations often raise questions regarding the relationship between TGA and vestibular symptoms. The following cases illustrate such representative examples.

### Representative cases

*Patient 1.* A 62-year-old woman with a previous history of benign paroxysmal positional vertigo (BPPV) presented to the emergency department with vertigo that had developed upon awakening in the morning. In the emergency room, she repeatedly asked “Where am I?” and “Why and how did I come here?” On examination, she was alert, but could not recall any event during the previous day. She showed no spontaneous or gaze-evoked nystagmus. However, right Dix-Hallpike maneuver triggered paroxysmal clockwise torsional (upper poles of the eyes beating to the right ear) and upbeat nystagmus along with vertigo, which was consistent with BPPV involving the right posterior canal (PC). Bedside head impulse tests were normal for all six semicircular canals. Other findings of neurological examination were normal. DWI one and a half hours after awakening (vertigo onset) was normal. Upon admission 5 h after awakening, she began to show a recovery of her memory, but still could not recall the events from awakening on that day. The positional vertigo resolved with the Epley maneuver for right PC-BPPV. Follow-up MRIs 3 days later revealed a focal high signal intensity in the body of right hippocampus. She showed visuospatial dysfunction during the clock drawing and the Rey Complex Figure Test (RCFT). Electroencephalography was normal.

**Fig. 1:**
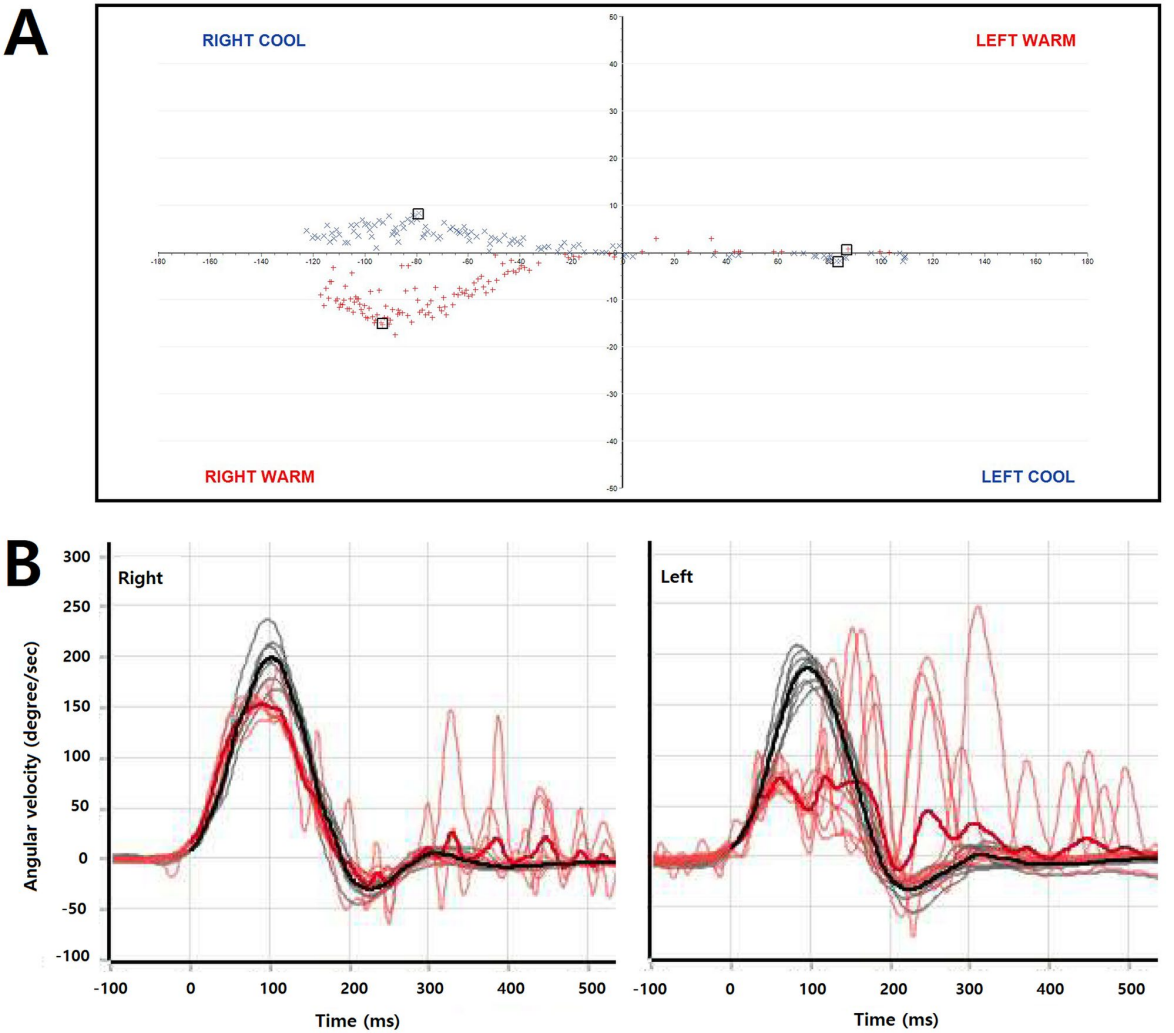
Caloric test (**A**) and video head impulse test (**B**) in patient 2. The caloric tests documented a left canal paresis at 80% and video head impulse test revealed a decreased gain (0.57) with covert and overt saccades for the left horizontal canal

*Patient 2.* A 58-year-old otherwise healthy man presented with disorientation that had developed just after a shower. The patient had been well until 5:30 p.m. on that day, but could not remember the events from 6:30 p.m. In the emergency department, he asked the same questions and did not know why and how he came to the hospital. He was alert, but disoriented to time, including the day and year. He could not recall several episodes during the previous three days. DWI two hours after symptom (disorientation) onset was normal. Although he showed a recovery of orientation and memory after admission, his recent memory remained impaired for the events after the shower. Further examination on admission showed spontaneous right beating nystagmus (2˚/s) without visual fixation, but no gaze-evoked or positional nystagmus. The video-head impulse tests (vHITs) were positive for the left horizontal and posterior canals and caloric tests documented a left canal paresis at 80% (Fig. 1). These findings were mostly consistent with acute unilateral peripheral vestibulopathy/vestibular neuritis involving the left side. Follow-up MRIs 2 days later disclosed a lesion on the tail of the left hippocampus.

**Fig. 2:**
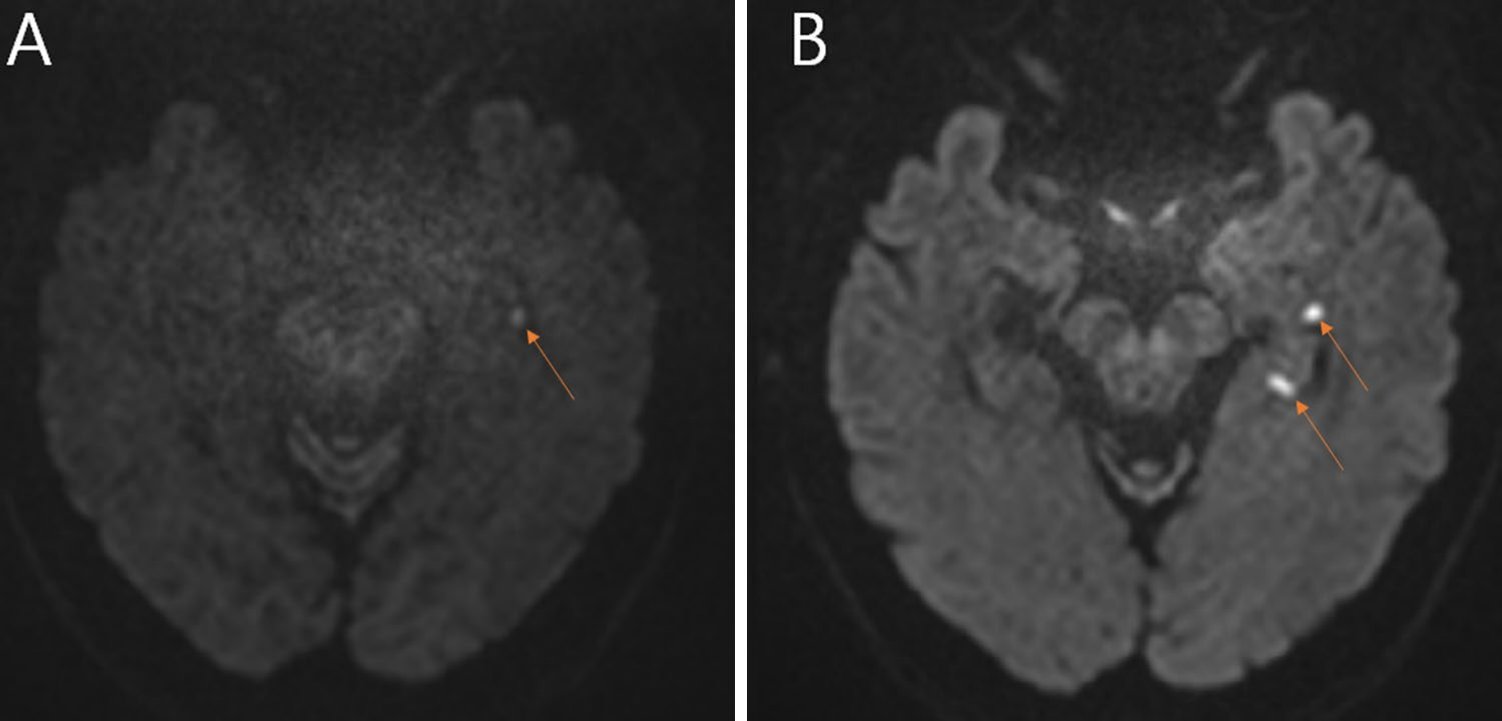
DWI in patient 3. The initial DWI showed a subtle high signal intensity involving the left hippocampal head (**A**). Follow-up DWI revealed two distinct lesions involving the head and body of left hippocampus (**B**)

*Patient 3*. A 67-year-old woman with dyslipidemia and hypothyroidism experienced a brief episode of vertigo while cleaning her house in the morning of the previous day. The next morning, she visited the emergency department due to recurred vertigo. In the emergency department, she repetitively asked why she was there for about 4 h. Even after a recovery of vertigo, she could not remember the events during the previous 4 h. In addition, she had headache, dizziness, nausea, and vomiting. She denied any specific stressful events around the onset of symptoms. The dizziness was continuous and worsened on lying down or turning in bed. Headache was of a pressing quality in the frontal area. Examination showed left beating spontaneous nystagmus without visual fixation, which was interrupted by right beating nystagmus for about 5 s. The left beating nystagmus persisted thereafter for more than 3 min without a direction reversal. Rightward smooth pursuits (SP) was impaired. Findings of vHITs and caloric tests were normal. Cervical vestibular-evoked myogenic potentials (VEMPs) showed a decreased amplitude during stimulation of the left ear and ocular VEMPs were normal. The initial DWI one hour after the vertigo onset showed a subtle high signal intensity in the left hippocampal head (Fig. 2A). Follow-up DWI revealed two distinct lesions involving the head and body of left hippocampus (Fig. 2B). Echocardiography was normal, but Holter monitoring revealed several episodes of non-sustained atrial tachycardia.

Motivated by these clinical observations, we aimed to systematically investigate the prevalence and characteristics of dizziness and spatial disorientation in patients with TGA, and to explore their potential association with hippocampal lesions and vestibular pathway abnormalities.

## Subjects and methods

### Patients and diagnostic criteria

We had recruited 64 TGA episodes from 63 patients [45 (71.4%) women, mean age ± SD = 58.6 ± 9.4] at the department of neurology of Korea University Ansan Hospital from May 2016 to April 2019. Detailed features of the episodes were obtained from the patients and/or the witnesses during the attacks. The information included amnesic duration, precipitating factors, preceding activities which were classified into six categories (emotional stress, stressful physical activity, water immersion, loud speech, travel, and sexual intercourse), accompanied symptoms such as headache, dizziness and disorientation during the amnesic periods, presence of vascular risk factors or neuropsychiatric disorders, and other comorbidities. The presence of dizziness could not be identified in two patients. The presence of spatial disorientation was determined based on two clinical features: (1) “Whether the patient experienced events during TGA episodes- such as getting lost or being unable to navigate familiar environments- that could not be solely attributed to memory impairment;” (2) “Whether episodes of disorientation occurred after hospitalization, such as difficulty locating their hospital room or the bathroom within it.”

The clinical diagnosis of TGA was made according to the criteria proposed by Caplan and Hodges [8], which included (1) The attacks must be witnessed. (2) There must be anterograde amnesia during the attack. (3) Cognitive impairment is limited to amnesia. (4) No clouding of consciousness or loss of personal identity. (5) No focal neurological sign or symptoms. (6) No epileptic features. (7) The attacks must resolve within 24 h. (8) No recent head injury or active epilepsy. And patients with a prior diagnosis of dementia, history of vestibular disorders, use of vestibulotoxic medications (e.g., gentamicin, vincristine), or severe medical conditions (e.g., hepatic, renal, or malignant diseases) were excluded.

### Brain imaging

DWI with the protocol of B1000 or B2000 was arranged immediately after the clinical diagnosis of TGA based on the interview. The median interval from symptom onset to MR imaging was 7.0 h (range = 0.5–96 h). The patients also had follow-up MRIs two days later with a protocol that included DWI, T1- and T2-weighted, fluid-attenuated inversion recovery, gradient echo images, and MR angiography with or without gadolinium enhancements.

### Neuropsychological assessments

Neuropsychological assessments included the Korea-Mini Mental Status Examination (K-MMSE) [9], the Korean version of the Montreal cognitive assessment (K-MoCA) [10], and a language function test. Cognitive function was evaluated in 58 participants (59 cases) during the post-acute phase using the Consortium to Establish a Registry for Alzheimer’s Disease-Plus battery. This battery included the subtests for attention, psychomotor speed and flexibility, executive function, visuoperceptual and visuoconstructive function, and memory [13]. The absolute values of the following subtests were included at the group level: word-list learning (immediate, delayed recall and discrimination), figural learning (delayed recall), and the Trail Making Test Part B; these were considered the parameters of verbal learning, nonverbal learning, and executive function. Specifically, the presence of spatial disorientation was evaluated with the interlocking pentagon drawing, the clock drawing test, and copying the RCFT. Behavioral and psychological symptoms were evaluated using the Korea version of the neuropsychiatric inventory. The K-MMSE and RCFT were scored using the Seoul Neuropsychological Screening Battery-II computerized scoring system, with cognitive impairment defined as a Z-score below –1.0 based on age- and education-adjusted norms. The K-MoCA was assessed using an age- and education-adjusted cutoff value.

### Ocular motor and vestibular function tests

Sixty patients had evaluation of the ocular motor and vestibular function using the video-oculographic recording (SLVNG, SLMED, South Korea) of spontaneous nystagmus (SN), head-shaking nystagmus (HSN), positional nystagmus (PN), horizonal SP, horizontal saccades, and vHITs. Bithermal caloric tests and ocular and cervical VEMPs were also performed. The severity of dizziness was assessed using the dizziness handicap inventory (DHI). All these evaluations were performed within a week after symptom onset. For each test, detailed methods and criteria for abnormalities have been described previously [11–15].

### Statistical analysis

We used Chi-square test or Fisher’s exact test to analyze the categorical variables and used Wilcoxon rank sums test for the continuous variables. Logistic regression analyses were adopted to assess the factors associated with dizziness and other factors. All statistical analyses were conducted using R 3.6.1 statistical computing language (R Foundation for Statistical Computing, Vienna, Austria).

### Standard protocol approvals, registrations, and patient consents

The institutional review board approved the retrospective nature of study protocols with an exemption of the patient consent (IRB number: AS16151-002). Written informed consent was waived by the board due to the retrospective design and the use of de-identified data, in accordance with institutional and national regulations.

## Results

### Clinical features

**Fig. 3:**
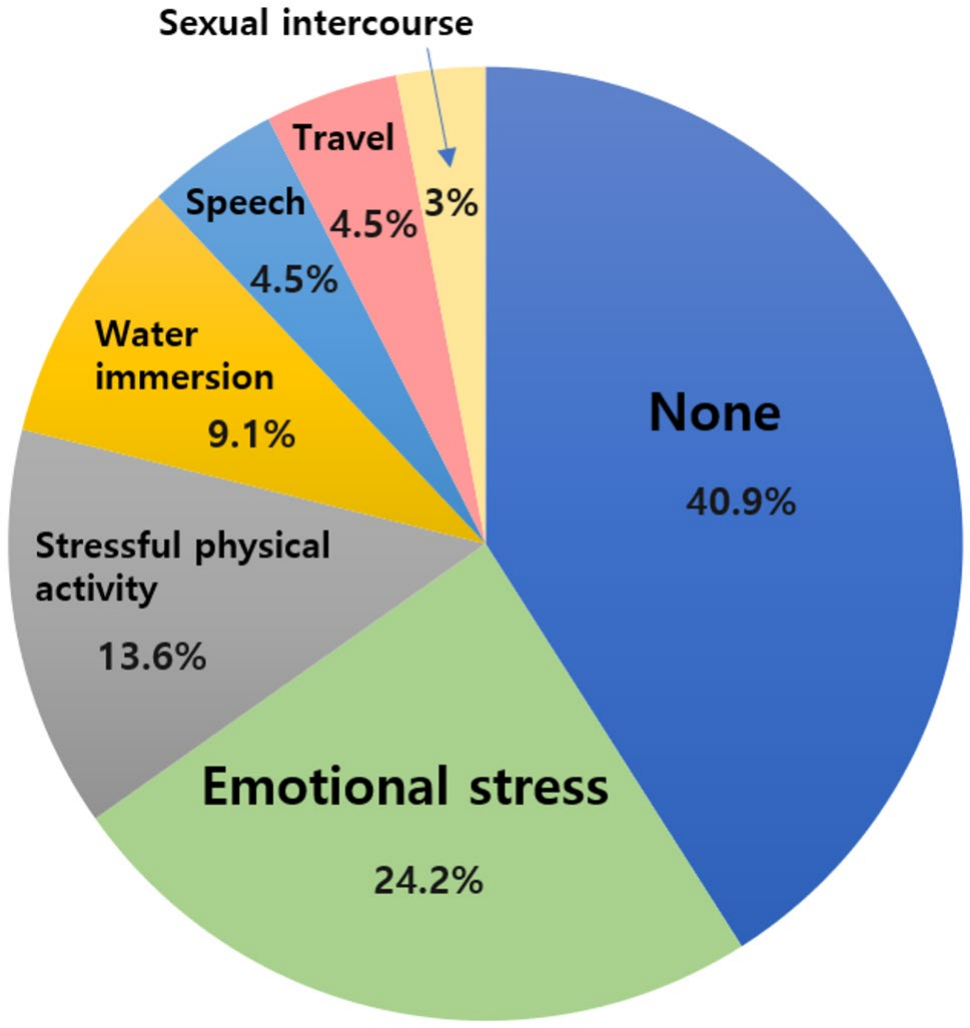
Preceding events in TGA episodes

The duration of amnesia was reported from 10 min to 23 h (median = 5 h). During the attacks, patients experienced dizziness in 19 of 62 episodes (30.6%), headaches in 18 of 64 episodes (28.1%), spatial disorientation in 16 of 64 episodes (25.0%). Patients mostly reported physical or psychological stressful events just before the onset of amnesia (51/64, 79.7%), which included emotional stress (24.2%), stressful physical activity (13.6%), water immersion (9.1%). Among these, about a half of the events were considered to have possibly provoked the Valsalva maneuver (50.0%) (Fig. 3).

One or more vascular risk factors were present in 27 (43.5%) patients (Table 1). Hypertension, neuropsychiatric disorders, and headache were more commonly found in women than in men. Univariable regression identified headache [Odds ratio (OR) = 7.07, *p* = 0.002], Valsalva maneuver (OR = 4.28, *p* = 0.017), and abnormal ocular VEMPs (OR = 4.00, *p* = 0.031) as significant predictors of dizziness. Multivariable logistic regression using backward elimination confirmed that headache [OR = 6.94, 95% Confidence interval (CI) 1.91–25.25, *p* = 0.003] and Valsalva maneuver (OR = 4.19, 95% CI 1.14–15.38, *p* = 0.031) remained independently associated with dizziness (Table 2). Additionally, multivariable regression using backward elimination indicated a trend toward an independent association between abnormal cervical VEMPs and spatial disorientation (OR = 3.28, 95% CI 0.885–12.20, *p* = 0.076) (Table 3).

**Table 1:** Demographic characteristics of patients and clinical features associated with dizziness in TGA episodes

Demographic characteristics	Patients without dizziness, n (42 patients)	Patients with dizziness, n (19 patients)	*p*-value
Woman (%)	29 (69.0%)	15 (78.9%)	0.425
Age	59.3 ± 10.1 (years)	57.8 ± 8.2 (years)	0.697
Hypertension	10 (23.8%)	7 (36.8%)	0.293
Dyslipidemia	6 (14.3%)	4 (21.1%)	0.710
Diabetes mellitus	2 (4.8%)	2 (10.5%)	0.582
Cancer	3 (7.1%)	0 (0)	0.546
Neuropsychiatric disease	2 (4.8%)	1 (5.3%)	1.000
Cerebrovascular accidents	1 (2.4%)	1 (5.3%)	0.530
Anemia	2 (4.8%)	0 (0)	1.000
Autoimmune disease	0	1 (5.3%)	0.312

Clinical presentation (62 episodes)	Without dizziness, n (43 episodes)	With dizziness, n (19 episodes)	
Provocation present	28 (65.1%)	10 (52.6%)	0.352
Valsalva like stress	17 (39.5%)	14 (73.7%)	0.013
PFO	16 (38.1%)	8 (47.1%)	0.526
Headache	7 (16.3%)	11 (57.9%)	0.001
Spatial disorientation	11 (25.6%)	3 (15.8%)	0.519
Amnesia duration	6.3 ± 4.7(hours)	6.3 ± 3.6 (hours)	0.685

Presence of dizziness could not be identified in two patients

Episodes include multiple TGA events in the same patient

Categorical variables were analyzed using the Chi-square test or Fisher’s exact test

Continuous variables were analyzed the Wilcoxon rank-sums test

*PFO* patent foramen ovale

**Table 2:** Odds ratios for dizziness in TGA episodes

Factors	Univariable						Multivariable					
	OR		95% CI		*p*-value		OR	95% CI			*p*-value	
Sex (woman)	1.625	0.452		5.848		0.457						
Age	0.984	0.930		1.042		0.585						
Hypertension	1.925	0.597		6.204		0.273						
Dyslipidemia	1.644	0.405		6.670		0.486						
Diabetes mellitus	2.412	0.314		18.546		0.398						
CVA	2.333	0.138		39.392		0.557						
Psychiatric disorders	1.139	0.097		13.378		0.918						
PFO	1.444	0.463		4.507		0.526						
Headache	7.071	2.091		23.918		0.002	6.941		1.909	25.245		0.003
Amnesia duration	0.999	0.883		1.131		0.990						
Spatial disorientation	0.545	0.133		2.236		0.400						
Provocation event	0.595	0.199		1.784		0.354						
Valsalva	4.282	1.303		14.078		0.017	4.186		1.139	15.380		0.031
CVEMPs	0.856	0.280		2.614		0.784						
OVEMPs	4.000	1.135		14.093		0.031						

Multivariable logistic regression was performed using backward elimination. All candidate variables were initially included, and non-significant variables were sequentially removed based on a significance level of 0.05

*CVA* cerebrovascular accident, *PFO* patent foramen ovale, *OVEMPs* ocular vestibular-evoked myogenic potentials, *CVEMPs* cervical vestibular-evoked myogenic potentials

**Table 3:** Odds ratios for spatial disorientation in TGA episodes

Factors	Univariable						Multivariable					
	OR		95% CI		*p*-value		OR	95% CI			*p*-value	
Sex (woman)	0.817	0.238		2.807		0.748						
Age	0.999	0.940		1.061		0.969						
Hypertension	1.364	0.394		4.725		0.625						
Dyslipidemia	0.714	0.135		3.774		0.692						
Diabetes mellitus	2.412	0.314		18.546		0.398						
CVA	infinity					1.000						
Psychiatric disorders	infinity					1.000						
PFO	0.650	0.192		2.205		0.489						
Headache	0.308	0.062		1.532		0.150						
Amnesia duration	1.052	0.928		1.193		0.426						
Dizziness	0.545	0.133		2.236		0.400						
Provocation event	0.771	0.245		2.431		0.658						
Valsalva	1.970	0.617		6.286		0.252						
CVEMP	2.859	0.819		9.982		0.100	3.284		0.885	12.195		0.076
OVEMP	0.773	0.184		3.249		0.725						

Multivariable logistic regression was performed using backward elimination. All candidate variables were initially included, and non-significant variables were sequentially removed based on a significance level of 0.05

### Radiologic findings

**Fig. 4:**
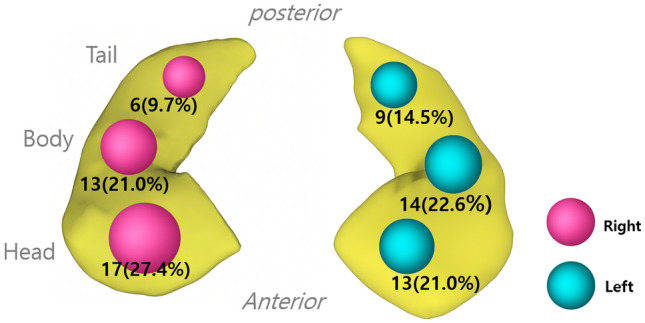
Location and proportion of hippocampal lesions in patients with TGA. 3D-rendered hippocampal models show lesion locations segmented into head, body, and tail regions in total of 48 cases

Acute lesions were identified on the initial DWI in 28 out of 63 cases (45.2%) and on follow-up MRIs in 45 cases (73.8%). Overall, acute MRI lesions were found in 48 (75.0%) cases with a hippocampal involvement in all of these cases. The hippocampal lesions were located on the left side in 16 (33.3%), on the right side in 13 (27.0%), and on both sides in 17 (35.4%). The lesions were considered to involve the head in 31 (42.4%), the body in 26 (35.6%), and the tail in 16 (21.9%) patients (Fig. 4). Extrahippocampal lesions were found in four with scattered lesions in two, a right frontal lesion in one, and a right cerebellar lesion in the remaining one. On MRA, three patients showed vertebral artery dissection, and another had bilateral stenosis of the posterior cerebral artery (PCA). The lesion side and location did not correlate with the presence of dizziness (*p* = 0.15) (Table 4).

**Table 4:** Statistical comparison of radiologic findings in TGA episodes with and without dizziness

Hippocampal lesion		Total (%)	Episodes without dizziness, n (%)	Episodes with dizziness, n (%)	*p*-value
Left head		13 (21)	9 (20.9)	4 (21.1)	1.000
Left body		14 (22.6)	7 (16.3)	7 (36.8)	0.102
Left tail		9 (14.5)	6 (14)	3 (15.8)	1.000
Right head		17 (27.4)	12 (27.9)	5 (26.3)	0.897
Right body		13 (21)	9 (20.9)	4 (21.1)	1.000
Right tail		6 (9.7)	6 (14)	0 (0)	0.165
Head (left or right)		23 (37.1)	16 (37.2)	7 (36.8)	0.978
Body (left or right)		22 (35.5)	13 (30.2)	9 (47.4)	0.194
Tail (left or right)		14 (22.6)	11 (25.6)	3 (15.8)	0.519
Initial lesion		28 (45.2)	21 (48.8)	7 (36.8)	0.382
Follow up lesion		45 (73.8)	32 (76.2)	13 (68.4)	0.543
Lesion side	Left	15 (24.2)	8 (18.6)	7 (36.8)	0.154
	Right	13 (21)	12 (27.9)	1 (5.3)	
	Both	18 (29)	12 (27.9)	6 (31.6)	

Based on 48 patients with hippocampal lesions

Categorical variables were analyzed using chi-square test or Fisher’s exact test

Continuous variables were analyzed using Wilcoxon rank-sum test

### Ocular motor findings

Nystagmus was observed in 32 patients (53.3%), spontaneous in six (10.3%), head- shaking in nine (15.5%), and positional in 24 (41.4%). SP was impaired in 39 patients (67.2%), bilaterally in 31 (53.4%), leftward only in five (8.6%), and rightward only in three (5.3%). In contrast, saccades were abnormal only in six patients (10.3%), including hypometria in five and slowing of rightward saccades in one.

The results of vHITs were abnormal in six (10.3%) patients. Asymmetric or unilateral caloric paresis was found in nine (15.8%) patients with a decreased response during left ear stimulation in all of them. Cervical VEMPs were abnormal in 25 (44.6%) of the 58 patients tested; decreased amplitude during left ear stimulation in 14 (25.0%) and during right ear stimulation in 10 (17.9%), and no responses on both sides in the remaining one. Ocular VEMPs were also abnormal in 14 (24.1%) of 58 patients evaluated; decreased amplitude during right ear stimulation in eight (13.8%) and during left ear stimulation in six (10.3%). The association between dizziness and vestibular test findings was not statistically significant in most categories, there was a trend toward significance for abnormal ocular VEMPs in patients with dizziness (42.1% vs. 15.4%, *p* = 0.047) (Table 5).

**Table 5:** Statistical comparison of vestibular function abnormalities in TGA episodes with and without dizziness

		Total (%)	Episodes without dizziness, n (%)	Episodes with dizziness, n (%)	*p*-value
SN		6 (10.3)	3 (7.7)	3 (15.8)	0.382
HSN		9 (15.5)	6 (15.4)	3 (15.8)	1.000
Positional N		24 (41.4)	15 (38.5)	9 (47.4)	0.518
SP		39 (67.2)	27 (69.2)	12 (63.2)	0.068
Saccades		6 (10.3)	4 (10.3)	2 (10.5)	1.000
Left HIT-HC		5 (8.6)	3 (7.7)	2 (10.5)	1.000
Left HIT-AC		0 (0)	0 (0)	0 (0)	-
Left HIT-PC		3 (5.2)	2 (5.1)	1 (5.3)	1.000
Right HIT-HC		1 (1.7)	0 (0)	1 (5.3)	0.328
Right HIT-AC		0 (0)	0 (0)	0 (0)	-
Right HIT-PC		1 (1.7)	0 (0)	1 (5.3)	0.328
Caloric test	Normal	48 (84.2)	34 (89.5)	14 (73.7)	0.143
	L paresis	9 (15.8)	4 (10.5)	5 (26.3)	
	R paresis	0 (0)	0 (0)	0 (0)	
CVEMP		25 (44.6)	17 (45.9)	8 (42.1)	0.784
OVEMP		14 (24.1)	6 (15.4)	8 (42.1)	0.047

Categorical variables were analyzed using the chi-square test or Fisher’s exact test

Continuous variables were analyzed using the Wilcoxon rank-sums test

*SN* spontaneous nystagmus, *HSN* head-shaking nystagmus, *Positional N* Positional nystagmus, *SP* smooth pursuits, *HIT* head-impulse test, *HC* horizontal canal, *AC* anterior canal, *PC* posterior canal, *L* left, *R* right, *OVEMP* ocular vestibular-evoked myogenic potentials, *CVEMP* cervical vestibular-evoked myogenic potentials

### Neuropsychological tests

The average K-MMSE score was 27.7 (SD = 2.3), with 9 out of 59 patients (15.3%) exhibiting abnormal results. The mean K-MoCA score was 23.9 (SD = 3.4), and 10 (16.9%) patients showed abnormal performance. Abnormalities in language function were identified in four patients (6.8%). Visuoperceptual and visuoconstructional deficits, assessed via interlocking pentagon and clock-drawing tests, were observed in 27 patients (45.8%). Executive dysfunction was also common, with 27 patients (46.6%) showing impairment on frontal/executive domain tasks.

When stratified by clinical features, patients with dizziness showed higher prevalence of abnormal K-MMSE (26.3% vs. 10.5%) and K-MoCA (26.3% vs. 13.2%) scores, though these differences were not statistically significant (*p* = 0.143 and 0.275, respectively). Language impairment was also more frequent in the dizziness group (15.8% vs. 2.6%, *p* = 0.103). In contrast, patients with spatial disorientation tended to have lower tendency for abnormal global cognition, with only 1 out of 16 (6.3%) showing abnormal K-MoCA results.

With regard to visuospatial memory, immediate recall impairment on the RCFT was present in 27 patients (47.4%), and a trend was found in the spatial disorientation group, which showed less frequent impairment (26.7% vs. 54.8%, *p* = 0.061). Delayed recall and recognition scores were abnormal in 54.4% of patients, regardless of symptom subgroup.

At follow-up, cognitive function became normal in most patients who initially showed abnormalities: 7 of 9 patients with abnormal K-MMSE, 6 of 10 with abnormal K-MoCA, and 1 of 4 with language impairment demonstrated a recovery. These findings suggest that while transient cognitive deficits are common in the acute stage of TGA, they are often reversible.

## Discussion

### Dizziness and TGA: causal relationship

As illustrated by the representative cases, it remains uncertain whether dizziness is a cause or a consequence of TGA. This ambiguity arises because the core feature of TGA—anterograde and retrograde amnesia—limits the patient’s ability to recall events around the time of onset. In cases such as BPPV, severe vertigo may induce emotional stress [16], suggesting that dizziness itself could serve as a preceding trigger. The presence of VEMP abnormalities in TGA patients may indicate an underlying vestibular vulnerability that predisposes individuals to vertigo attacks.

However, a previous study on hippocampal lesions presenting with acute vestibular syndrome reported that all patients experienced vertigo, with or without amnesia [17], raising the possibility that vestibular dysfunction may also result from TGA or associated hippocampal lesion.

### Dizziness and spatial disorientation in TGA

In our patients with TGA, dizziness and spatial disorientation were observed in 30.6% and 20.5% of patients, respectively. Previous studies have also noted dizziness as a frequent complaint in patients with TGA or hippocampal infarction [17, 18]. Indeed, dizziness or spatial disorientation was reported in 40% of patients with the lesions involving the hippocampal area [17]. Notably, disorientation was found more often in TGA than in isolated hippocampal infarction [17].

Electrophysiological studies have demonstrated a strong interaction between the vestibular system and the hippocampus, suggesting that the hippocampus plays a compensatory role in vestibular dysfunction arising from either peripheral or central causes [19]. Neurons in the vestibular nuclei project to the visual and parietal cortices, as well as to the thalamus, a key relay station in the ascending vestibular pathways [20, 21]. The hippocampus receives vestibular inputs via the perirhinal and entorhinal cortices [22, 23]. Head direction cells in the anterior thalamic nuclei may convey the vestibular information to the hippocampus through the postsubiculum and entorhinal cortex [24]. Some hippocampal neurons have also been shown to respond to linear and angular motion, as well as to optokinetic stimuli [25], indicating integration of vestibular inputs during hippocampal processing. Moreover, hippocampal place cells are known to modify their firing patterns based on location [26].

During navigation tasks, patients with TGA exhibit higher error rates in allocentric spatial orientation during the post-acute phase compared to healthy controls [27]. These patients tend to rely more on visual cues, as evidenced by increased fixation on salient landmarks during allocentric navigation [27]. These findings align with the persistent spatial orientation deficits observed in our study. Since vestibular dysfunction can result in behavioral impairments similar to those caused by hippocampal damage, the vestibular system likely modulates hippocampal function and supports spatial information processing [28–30]. However, this neural interaction does not fully account for why disorientation occurs more frequently in TGA than in hippocampal infarction without amnesia [2, 17]. It is possible that TGA reflects a state of acute focal metabolic stress, which may not necessarily be ischemic in nature [16].

Previous neuroimaging studies have demonstrated hypometabolism in the medial temporal lobe during both the acute and post-acute stages of TGA, supporting the role of hippocampal dysfunction in this disorder [31, 32]. In the post-acute phase, patients with TGA show decreased activity not only in the hippocampus, but also in the posterior parietal and prefrontal cortices, as well as in the cerebellum [33]. This pattern of hypometabolism may underlie the visuospatial deficits associated with TGA.

### Dizziness, comorbidities, and precipitating activities

To identify clinical characteristics associated with dizziness in patients with TGA, we compared demographic and clinical variables between patients with and without dizziness (Table 1).

Patients with TGA frequently report a preceding event, many of which are activities associated with a Valsalva maneuver [34, 35]. These include emotional stress, water immersion, or intense physical activity such as sexual intercourse. In our study, such events were reported in 50.0% of patients. The Valsalva maneuver affects cardiac output and can cause transient cerebral hemodynamic changes, resulting in symptoms such as lightheadedness or dizziness [36]. It may also increase intracranial or tympanic pressure, further contributing to vestibular symptoms. Of interest, however, provoking events were not significantly associated with dizziness (*p* = 0.352) although those were common (65.1% without dizziness vs. 52.6% with dizziness).

In a prior study on TGA, most patients presented with nausea or vertigo, while only a subset exhibited disorientation or memory impairment [17]. However, it remained unclear whether vertigo was a triggering factor or a symptom experienced during or after the amnesic episode. In contrast, vertigo preceded the onset of amnesia in three representative cases of our study. Two were diagnosed with benign paroxysmal positional vertigo (BPPV) and acute unilateral vestibulopathy, respectively, while the third showed alternating nystagmus during the attack. Furthermore, a previous study on hippocampal lesions presenting with acute vestibular syndrome reported that all patients experienced vertigo, with or without amnesia [17]. These findings support the notion that both peripheral and central vestibulopathies may be associated with hippocampal dysfunction and the clinical manifestation of TGA.

Taken together, these results suggest that dizziness in TGA is not merely an epiphenomenon but may reflect a complex interplay between vestibular input, vascular dynamics, and hippocampal susceptibility—particularly in the context of Valsalva-like physiological stress.

Of note, headache was reported in 29.0% (18/62) of TGA episodes in our cohort, and was significantly more common in patients with dizziness than in those without (57.9% vs. 16.3%, *p* = 0.001). Although headache was not significantly associated with spatial disorientation, this strong relationship with dizziness suggests a potential overlap with migraine-related mechanisms. Previous studies have suggested that a subset of TGA may be linked to migraine, either as a comorbidity or as a possible manifestation of a migrainous process [37]. For instance, Arena et al. reported that 42% of patients with TGA had a personal history of migraine, and proposed that TGA could represent a rare form of migraine aura in susceptible individuals. Vestibular migraine, in particular, is known to present with episodic vertigo, dizziness, and even transient amnesia in some cases, mediated by transient cortical spreading depression or brainstem-hypothalamic dysfunction. Given this background, our finding of the significant association between headache and dizziness during TGA episodes may support a shared pathophysiology involving transient hemodynamic or neurovascular dysregulation. However, since detailed migraine history was not systematically assessed in our cohort, further prospective studies are warranted to clarify the relationship between TGA, migraine, and vestibular dysfunction.

### Ocular motor and vestibular function in TGA patients with dizziness

To date, no studies have systematically evaluated the prevalence of dizziness and characteristics of nystagmus during the acute and subacute stage of TGA. In this study, we could classify the vestibular dysfunction as peripheral, central, and undetermined. The vHITs and caloric tests are important for distinguishing peripheral from central lesions. In this study, the results of vHITs were abnormal in six (10.3%) patients, and asymmetric or unilateral caloric paresis was found in nine (15.8%) patients, all of whom showing a decreased response during left ear stimulation. In the peripheral group, three patients had vestibular hypofunction, two with left unilateral vestibulopathy and one with bilateral vestibulopathy, and another one had left PC-BPPV. In the central group, one showed alternating nystagmus, two had persistent positional upbeat nystagmus, and one exhibited persistent positional downbeat nystagmus with a leftward SP deficit.

Periodic alternating nystagmus (PAN), characterized by a conjugate horizontal nystagmus that periodically reverses direction, has been ascribed to the velocity storage mechanism. This mechanism may be associated with the activity of head direction (HD) cells, originally identified in the rat presubiculum [38], and subsequently localized to multiple regions within the Papez circuit, including the anterior dorsal thalamic nucleus [39], lateral mammillary nuclei [40], restrosplenial cortex [41], and entorhinal cortex [42], as well as the medial prestriate cortex and CA1 region of the hippocampus [43]. Damage to the hippocampus impairs the ability to maintain a consistent internal sense of direction when navigating novel environments [44], which is consistent with the result of previous studies, elevated allocentric spatial orientation errors in TGA patients [27], and with the persistent visuospatial deficits observed in our patients with TGA.

Although the association between dizziness and vestibular test findings was not statistically significant for most variables, there was a trend toward significance for abnormal ocular VEMPs in patients with dizziness (42.1% vs. 15.4%, *p* = 0.047). While this result did not remain significant in multivariable analyses, it suggests a potential vestibular contribution to dizziness symptoms in TGA. Previous research has suggested that otolithic dysfunction is linked to impaired spatial memory [45] and abnormal activity of head direction and place cells in the thalamus and hippocampus [46, 47]. In line with this, the visuoconstructive deficits observed in our TGA patients may be partially explained by otolithic dysfunction, as evidenced by the ocular VEMPs abnormalities.

### Cognitive function in TGA patients

Neuropsychological assessments in our cohort revealed that cognitive impairments during the acute phase of TGA are not limited to episodic memory, but also involve broader domains such as visuoconstructive function, frontal/executive processing, and spatial memory [48, 49]. Although not statistically significant, patients with dizziness exhibited a trend for abnormal K-MMSE and K-MoCA scores, as well as more frequent language dysfunction. These findings may indicate a more widespread cortical involvement or disruption of distributed cognitive networks in patients who experience dizziness during TGA [2, 31].

Of interest, patients with spatial disorientation demonstrated a lower frequency of immediate recall impairment on the Rey Complex Figure Test when compared to those without disorientation (26.7% vs. 54.8%, *p* = 0.061). While spatial disorientation might be expected to align with poor spatial memory, it is possible that the symptom reflects a transient navigational processing deficit rather than a failure in memory encoding [27]. Previous work has shown that the hippocampus, especially the CA1 subregion, plays a key role in encoding spatial memory and integrating multisensory inputs, including the vestibular signals [24, 26, 46].

Visuoconstructive and executive dysfunctions were frequently observed across all clinical subgroups, consistent with prior findings of persistent visuospatial deficits in TGA patients even after the resolution of amnesia [27]. These deficits are believed to arise from transient disconnection or dysfunction within the hippocampal–retrosplenial–parietal network, which supports spatial orientation and visuoconstructive processing [19, 28, 33].

The cognitive impairments observed during the acute phase mostly resolved over time, consistent with the transient and reversible nature of TGA [6, 16]. These patterns further support the hypothesis that TGA represents a functional disturbance of hippocampal-cortical networks, possibly triggered or modulated by vestibular or hemodynamic stressors [3, 33, 36]. However, the specific temporal profile of spatial disorientation—whether it represents a short-lived phenomenon confined to the acute phase or a more persistent disturbance—could not be clearly delineated in this study due to its retrospective design.

### Vestibulo-hippocampal interaction in TGA

The anatomical and functional connections between the vestibular system and hippocampus provide a plausible framework for understanding dizziness and spatial disorientation in TGA. Vestibular input reaches the hippocampus via multisynaptic pathways involving the vestibular nuclei, thalamus, retrosplenial cortex, and parietal cortices, as well as through theta-generating networks such as the medial septum and the entorhinal cortex [26, 50]. Animal studies have demonstrated that bilateral vestibular lesions lead to significant impairments in spatial memory and disrupt hippocampal place cell firing [40].

Functional MRI and lesion studies in humans further support this vestibulo-hippocampal link, with vestibular dysfunction associated with atrophy and altered connectivity in the hippocampus [46, 51]. In the context of TGA, acute dysfunction in either system may transiently impair spatial memory or orientation. Moreover, transient disruption in vestibular inputs may act as a stressor and precipitate hippocampal metabolic dysfunction. This explanation is consistent with the hypothesis the TGA results from a functional network disturbance. This dual-directional relationship may help explain why some patients experience vestibular symptoms, whereas, in others, hippocampal dysfunction may lead to disorientation or dizziness.

### Correlation with lesion location

Previous studies have reported that hippocampal lesions in TGA most frequently involve the body (41.7%), followed by the tail (33.3%) and head (25%) [17]. In our cohort, however, the most common lesion site was the head region of the hippocampus (37.1%), followed by the body (35.5%) and tail (22.6%) (Table 4). This distribution is more balanced than previously reported, and differences in imaging protocols or timing of DWI acquisition may account for these discrepancies.

We found no significant association between the lesion location and the presence of dizziness or spatial disorientation. For instance, hippocampal head lesions were equally distributed between the patients with and without dizziness (37.2% vs. 36.8%, *p* = 0.978), and spatial disorientation was not significantly associated with any particular location of lesions. Although the lesions involving the left hippocampal body appeared more frequent in patients with dizziness (36.8% vs. 16.3%) and spatial disorientation (37.5% vs. 18.8%), these findings did not reach a statistical significance.

These results are consistent with the current understanding of hippocampal functional anatomy. The anterior (head) portion of the hippocampus has been associated with vestibular processing and affective modulation, while the posterior (tail) region is more involved in visuospatial and mnemonic integration [5, 19]. The lack of clear localization effect in our findings suggests that TGA may reflect a functional network disturbance across the hippocampal regions rather than focal damage to a specific site.

## Conclusion

Patients with TGA frequently suffer from dizziness or spatial disorientation. Our findings suggest that these symptoms are not merely coincidental but may reflect transient dysfunction of specific vestibular pathways. In particular, dizziness was associated with otolith-ocular pathway dysfunction, while spatial disorientation was linked to vestibulo-spinal involvement. And clinical factors such as Valsalva maneuver activity and headache are risk factors for dizziness in TGA patients.

### Limitation of the study

The retrospective nature of this study may have resulted in potential biases, such as recall or selection bias from the patients and witnesses regarding the features and duration of amnesia. In addition, because of the exploratory design and relatively small sample size, we did not apply a correction for multiple comparisons (e.g., Bonferroni correction). This approach may increase the risk of false negative or false positive error, and therefore, the results with a marginal significance should be interpreted with caution. Moreover, the lack of long-term follow-up limits the assessment of the prognostic features of vestibular and neuropsychological dysfunction. The relatively small sample size of 63 patients also restricts expanding the findings to the broader TGA population, which necessitates larger studies for more definitive conclusions. Finally, the timing of MRIs varied significantly among the patients, ranging from 0.5 to 96 h after the symptom onset, which may have affected detection of hippocampal lesions and their correlation with the clinical symptoms.
